# Early Diagnosis of Parkinson’s Disease Through Lite HGWA-Net Model: A Hybrid CNN Based on Wavelet Transform and Attention Mechanism

**DOI:** 10.3390/diagnostics16040550

**Published:** 2026-02-13

**Authors:** Zohre Yaghoubi, Saeed Setayeshi, Sara Motamed, Malihe Sabeti

**Affiliations:** 1 Department of Computer Engineering, NT.C., Islamic Azad University, Tehran 13145, Iran; yaghoubi.z@iau.ac.ir (Z.Y.); sabeti@iau.ac.ir (M.S.); 2 Medical Radiation Engineering Department, Faculty of Energy Engineering and Physics, Amirkabir University of Technology (Tehran Polytechnic), Tehran 15916, Iran; setayesh@aut.ac.ir; 3 Department of Computer Engineering, FSh.C., Islamic Azad University, Fouman 43515, Iran

**Keywords:** Parkinson’s disease, T2-weighted MRI, deep learning, lightweight neural networks, wavelet transform, GhostNet, coordinate attention

## Abstract

**Background/Objectives**: Parkinson’s disease (PD) is a progressive neurodegenerative disorder in ageing populations, yet early diagnosis before motor symptoms remains critical. Reliable identification of subtle nigral alterations at early stages of the disease on magnetic resonance imaging (MRI) remains challenging. This limitation is mainly attributed to the subjective and low sensitivity of manual image interpretation in early PD. Here, we demonstrate a deep learning-based framework to enhance early PD detection. The study’s novelty is a lightweight deep learning framework that captures spatial, textural, and frequency-domain PD biomarkers without heavy network architectures or manual region delineation. **Methods:** The model integrates GhostNet with ensemble learning to combine local and global spatial information. This model employs wavelet-based frequency feature extraction rather than downsampling and incorporates an attention module to focus on relevant image regions, particularly changes in the substantia nigra (SN) region. Segmentation is employed solely as an auxiliary intermediate step to localize the SN and guide discriminative feature extraction. The final output is a binary classification that distinguishes PD patients from healthy controls. T2-weighted MRI data from the PPMI database are employed. **Results**: The proposed model achieved an F1-score of 0.8762, demonstrating robust performance under class imbalance, outperforming state-of-the-art models with only 2.03 million parameters and 4.36 Giga Floating Point Operations (GFLOPs). The architecture uncovered texture and frequency patterns previously inaccessible with conventional CNN pipelines. Model comparisons demonstrated consistent gains across all evaluated metrics (all *p* < 0.001), establishing robust diagnostic improvement. **Conclusions**: These findings establish an efficient, high-performing framework for reliable MRI-based PD identification. The approach provides automated early detection and supports clinically scalable, computationally lightweight screening tools.

## 1. Introduction

The accurate and early diagnosis of complex neurodegenerative disorders is a critical yet challenging task in clinical neuroscience. After Alzheimer’s, PD is the second most common neurodegenerative disorder, with a record of more than ten million worldwide [1]. The gradual loss of dopaminergic neurons in the substantia nigra, together with changes in the basal ganglia, is the cause of this disease. The available findings indicate that when approximately 60–80% of brainstem neurons are lost, motor symptoms like tremor, bradykinesia, and rigidity appear [2,3]. The pathological progression of PD begins years before this stage and is often accompanied by symptoms like sleep disturbances, depression, and olfactory dysfunction [4]. Since there is no definitive cure for PD and its progression can only be slowed by timely pharmacological interventions, identifying biomarkers in the early stages is critical [5].

**Figure 1:**
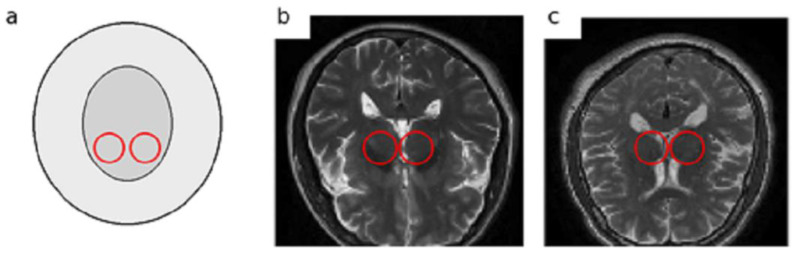
Representative T2-weighted sMRI examples with approximate SN localization: (**a**) schematic depiction of the substantia nigra (SN) region, (**b**) PD patient, and (**c**) healthy control. Highlighted circles indicate the approximate SN region.

In the early stages of PD, non-motor symptoms overlap with other neurological diseases like Multiple System Atrophy (MSA) or drug-induced Parkinsonism, the clinical diagnosis faces serious challenges [6,7]. These non-motor symptoms, including sleep disturbances, depression, olfactory dysfunction, and subtle cognitive changes, are often nonspecific and can lead to misdiagnosis or delayed diagnosis even by experienced clinicians [8]. MRI plays a vital role in assessing and monitoring structural and functional changes in the brain. It offers a non-invasive, safe, and cost-effective technique. T2-weighted MRI images, owing to their high contrast between grey and white matter and sensitivity to tissue changes, allow clear visualization of subcortical structures such as the SN and basal ganglia. These characteristics make T2-weighted MRI particularly suitable and widely employed in studies of PD [9]. Despite the potential of MRI for detecting structural brain changes, reliably identifying subtle nigral alterations in early-stage Parkinson’s disease remains highly challenging. Manual or semi-quantitative MRI assessments are prone to considerable diagnostic errors, reported to be ≥25. Furthermore, subtle structural changes in the substantia nigra and basal ganglia are often difficult to distinguish from normal ageing, resulting in significant inter-observer variability. These limitations highlight the need for automated, objective, and reproducible approaches capable of detecting fine-grained nigral changes that often escape human visual perception [10]. To better illustrate the anatomical target and the nature of T2-weighted structural MRI (sMRI) data used in this study, we provide representative examples with the approximate SN region highlighted Figure 1. Note that on conventional T2-weighted images, nigral alterations associated with early PD can be subtle and reader-dependent, motivating automated feature learning approaches.

The manual analysis of such a big and complex dataset requires high expertise. Because of the need for large-scale datasets, computational complexity, and limitations in extracting multiscale features, it remains challenging. This study introduces a lightweight and efficient framework that combines wavelets and attention mechanisms within a CNN architecture to extract both spatial and frequency features from MRI images. This method, in addition to reducing computational complexity, achieves higher accuracy in identifying changes in the substantia nigra. Although the methods adopted in PD diagnosis are practical and valuable, they inevitably have limitations, like reduced accuracy and stability. The focus here is on enhancing existing computational approaches and introducing a new solution to address these challenges and improve the disease-diagnosis rate.

In this study, we introduce a novel lightweight deep learning architecture that combines Hybrid Ghost modules, wavelet-based feature extraction, and coordinate attention to enable early detection of Parkinson’s disease from MRI. Unlike conventional CNN approaches, our model employs a wavelet-based downsampling strategy, which preserves both spatial and frequency-domain information critical for capturing subtle nigral changes. An auxiliary segmentation branch is incorporated to localize the substantia nigra and guide discriminative feature learning, while the primary task remains patient-level PD classification. The proposed framework achieves state-of-the-art diagnostic performance on the PPMI dataset, with significantly fewer parameters and lower computational cost compared to existing methods. Extensive experiments demonstrate the robustness, efficiency, and clinical potential of proposed model as a scalable tool for MRI-based Parkinson’s disease screening.

The structure of this article is as follows: literature review presented in Section 2; the method is explained in Section 3; the experiments and results are expressed in Section 4; the issue is discussed in Section 5; and the article is concluded in Section 6.

## 2. Literature Review

Early diagnosis of PD through MRI, specifically the T2-weighted, is gaining momentum. These images, owing to their ability to display structural changes in the substantia nigra, their non-invasive nature, and their lower cost relative to methods like PET or SPECT, are highly contributive [11]. In recent years, compared with traditional methods, deep learning models have improved the accuracy and efficiency of PD diagnosis [12]. Welton et al. achieved an AUC of approximately 94% by applying a DL and midbrain segmentation in MRI [13]. In comparison, Alrawis et al. achieved 97% accuracy on the PPMI dataset employing the FCN-PD method, which is based on a full CNN and EfficientNet [14]. Adding attention modules and hybrid architectures yields promising results. The findings suggest that combining CNNs with attention mechanisms can guide the network to critical brain regions, like the substantia nigra, and improve diagnostic accuracy [15]. The integration of Vision Transformer (ViT) with DenseNet and attention modules has enabled the identification of key MRI regions in the early stages of the disease [16]. Architectural advances, like the ConvKAN model [17], a combination of CNNs and Kolmogorov–Arnold Networks, have achieved an AUC of 0.99. Combining CNNs with metaheuristic algorithms, like Grey Wolf Optimization, or with multi-objective data improves accuracy [18]. Beyond PD, deep learning approaches have demonstrated strong potential for early diagnosis of other neurological disorders. Özdemir, Ş.N. and Yıldız, K. achieved high accuracy in early autism spectrum disorder detection using artificial neural networks and behavioural data [19]. ANN-based models using selected clinical and demographic features have shown promising performance in the early diagnosis of Alzheimer’s disease [20]. Table 1 provides an overview of recent studies on Parkinson’s disease diagnosis using MRI and deep learning models.

Recent trends in deep learning emphasize lightweight and hybrid architectures, which achieve a balance between high accuracy and low computational cost [21]. Models such as MobileNetV3 [22], ShuffleNet [23], and GhostNet [24] have been effectively applied in medical imaging due to their efficiency and ability to perform real-time inference, making them suitable for deployment in clinical environments.

Despite these advances, limitations such as the lack of large, labelled datasets and the interpretability of deep networks remain inevitable [25].

These limitations motivate this proposed model, Lite Hybrid Ghost Wavelet Attention Network (Lite HGWA-Net). This model achieves 98.24% accuracy on the PPMI data by combining a CNN, wavelets, and an attention module.

**Table 1:** Overview of the recent studies on PD with MRI and deep learning models.

The Studies (Year)	Dataset	Sample Size	Features	MRI	Model	Accuracy (%)
Yang et al. [26]	PPMI	800 MRI + SNP	MRI + Genetic Data	T1-weighted	Fusion CNN + SNP	96.7%
Li et al. [27]	Private	54 PD + 50 HC	MRI	Brain MRI	Deep learning-based classifier	Not reported
Islam & Khanam [28]	PPMI	567 MRI scans	Segmented GM/WM	T1-weighted	CNN on GM/WM	92.6%
Sahu & Chowdhury [29]	PPMI	356 PD	MRI + DTI (FA, MD)	T1 + DTI	CNN + Fusion	95.5%
Kollia et al. [30]	PPMI	391 PD + 167 Control	MRI + DaTscan	T1-weighted	TL-DNN + KNN	-
Tassew et al. [31]	PPMI IXI	900 PD + 900 Control	Two-dimensional slices of 3D T2-MRI	T2-weighted	Multi-channel Fusion CNN	97.2%
Dentamaro et al. [16]	PPMI	500 PD + 500 Control	MRI + Clinical Data	T1-weighted	DenseNet + Excitation Network	96.5%

Adopting deep architectures, particularly CNNs and their variants, yields high accuracy. Most studies report an accuracy rate of >92% highlight the pivotal role of deep architectures in improving diagnostic systems.

## 3. Materials and Methods

**Figure 2:**
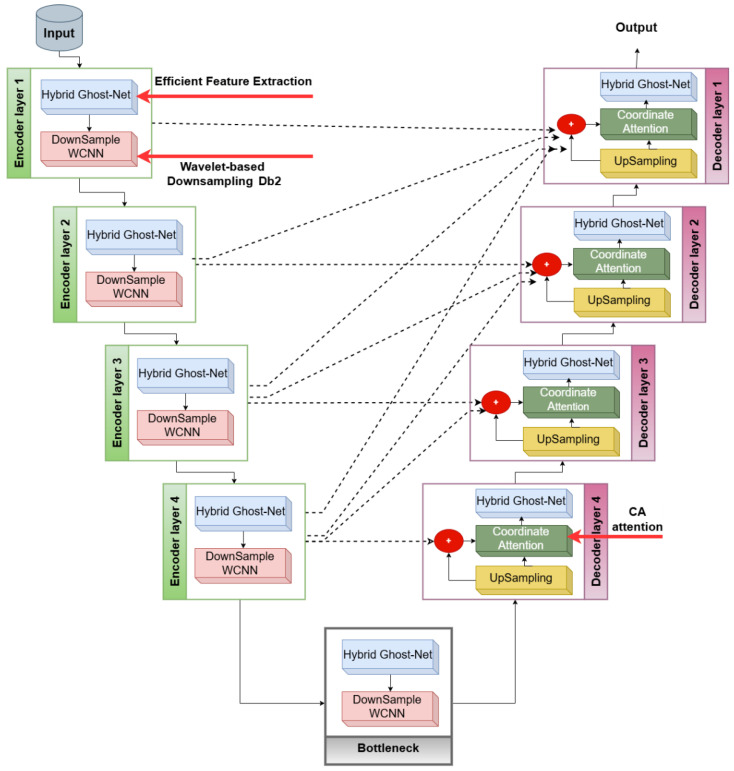
The overall architecture of Lite HGWA-Net consists of the data flow from input MRI images to the diagnostic output. The key components, Hybrid Ghost Modules for efficient feature extraction, WCNN blocks for wavelet-based downsampling, and a coordinate attention (CA), are marked with red arrows.

The study employs a quantitative experimental design. The architecture of the Lite HGWA-Net model (hybrid ghost wavelet attention), consisting of the Encoder, Wavelet-based CNN (WCNN), Attention Module, and Decoder sections with inter-track connections, is illustrated in Figure 2.

As observed in Figure 2, this model is a combination of Ghost modules, WCNN [32,33], and Attention Mechanisms, to increase the recognition accuracy while reducing the computational complexity. In the Encoder section, a combination of GhostNetV2 [34] and Learned Group Convolution (LGC) [35] is applied instead of standard convolutions. By generating additional features through lighter operations such as depthwise convolutions and inexpensive linear transformations, the Ghost modules would reduce the number of parameters while preserving the image’s spatial feature mapping. Their integration with LGC removes non-essential weights and optimizes the parameter space. To perform downsampling, rather than the conventional max-pooling operation in the U-Net, the wavelet transform is applied. In addition to preserving the image’s frequency and texture information, this choice, by removing fine details, prevents quality loss. Such an approach is essential in applications like PD diagnosis, where subtle changes in the substantia nigra (SN) and brain texture patterns are of great concern. In the Decoder section, the attention mechanism [36] and mid-scale skip connections are employed to enhance the model’s focus on critical regions, thereby optimizing multi-scale integration. This design improves the integration of high- and low-level information and maintains the model’s focus on relevant features. In practice, the modular structure of Lite HGWA-Net significantly reduces parameters and FLOPs while improving the accuracy of texture feature extraction related to neurodegenerative disorders. Although segmentation-based metrics are reported, the primary task of Lite HGWA-Net is patient classification for early diagnosis of Parkinson’s disease, with segmentation serving as an auxiliary mechanism to focus the analysis on the substantia nigra.

Lite HGWA-Net is designed for patient-level classification. An auxiliary segmentation branch predicts a binary SN mask to localize disease-relevant anatomy. The predicted mask is applied as an attention-like gating mechanism to intermediate feature maps (element-wise multiplication) to constrain feature learning to the SN region. The masked features are then aggregated and fed into fully connected layers to produce the final PD vs. healthy-control prediction.

### 3.1. Hybrid Ghost Model

**Figure 3:**
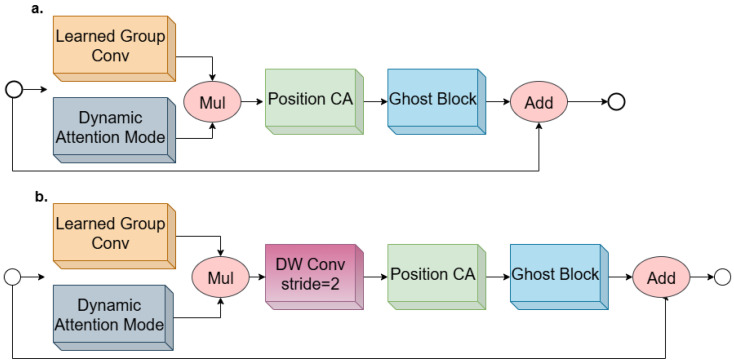
Architecture of the Hybrid Ghost module, where the GhostNetV2 is integrated with learned group convolution to reduce computational cost while maintaining strong representational capacity (**a**) with stride = 1; (**b**) with stride = 2.

In this design, the Hybrid Ghost module, Figure 3, is inspired by GhostNetV2 and dynamic learning groups to reduce computational burden while maintaining the ability to extract complex and valuable patterns. The Ghost module generates additional feature maps at a low cost, and the dynamic group convolution enables better adaptation to the data structure. This combination enables the network to extract both subtle and global patterns in brain images efficiently.

#### 3.1.1. Dynamic Learning Group Convolution (DLGC)

**Figure 4:**
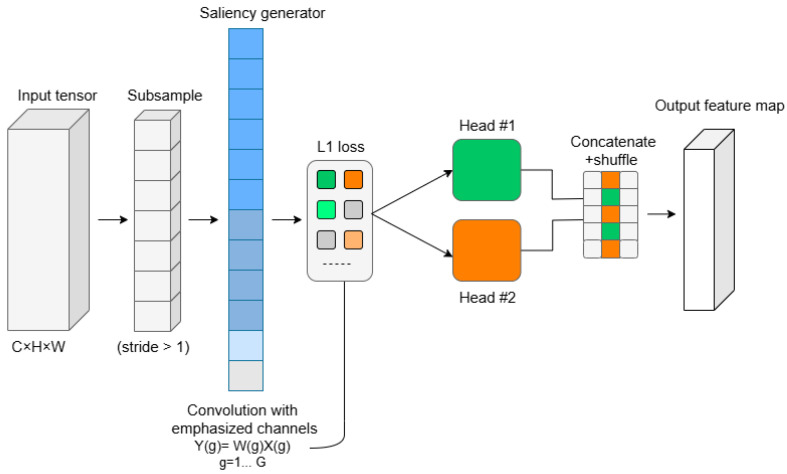
The general view of the learning group convolution method consists of a subsampling operation, a saliency channel selector (gate), group convolutions, and a combination of the outputs.

The DLGC technique is employed to reduce the number of parameters and computational load in CNNs [35]. As observed in Figure 4, in this method, the input is divided into many groups, and the convolution operation is applied independently to each group. Unlike classical group convolution, where the channel grouping is fixed [37], the learning version of the network learns and optimizes the channel grouping during training [35,36,37,38]. The basic group convolution is computed through Equation (1):

(1) Y(g)=W(g)×X(g) ,  g=1,2,…,G

where *G* is the number of groups, *X (g)* is the input to group g, *W (g)* is the weights, and *Y (g)* is the convolution output of each group.

This technique provides greater flexibility and enables the extraction of more complex and salient features. During training, in addition to learning the convolutional weights, the network employed a parameterization or modulation mechanism to determine channel grouping, which could be continuously adjusted to achieve optimal performance.

#### 3.1.2. Ghost Model Version 2: Combining Efficiency and Long-Range Dependency Modelling

**Figure 5:**
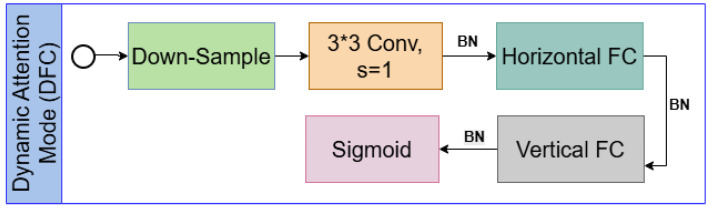
The horizontal and vertical correlations from the global pixel connections with low computational cost, by combining the local and global information.

GhostNetV2 is developed to model long-range dependencies. While GhostNetV1 [24] reduces computational cost via lightweight operations such as depthwise convolutions, its limited spatial interaction restricts the network’s expressive power. GhostNetV2 generates intrinsic feature maps using standard convolutions and additional ghost feature maps through lightweight operations such as depthwise convolutions and inexpensive linear transformations enabling efficient feature representation. These lightweight operations generate additional feature maps with minimal computational overhead, which explains how GhostNetV2 achieves parameter efficiency while maintaining expressive power. By introducing the decomposed Fully Connected Attention (DFCA) mechanism, the horizontal and vertical correlations from global pixel connections are extracted at low computational cost, and local and global information are combined to identify complex patterns in brain images, Figure 5.

### 3.2. Wavelet-Based CNN

**Figure 6:**
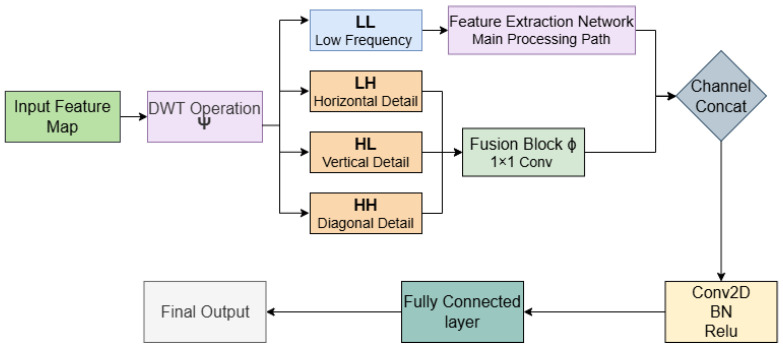
Architecture of the wavelet-based convolutional neural network (WCNN).

The dimensionality reduction in the classical U-Net is replaced with a wavelet-based method to preserve both spatial and frequency information in MRI images. As observed in Figure 6, unlike MaxPooling, the discrete wavelet transform (DWT) divides the input feature map X ∈ R^ (H × W) into four sub-bands by applying Equation (2):

(2) {LL,LH,HL,HH}=Ψ(X)

where *LL* is the overall image information, and the high-frequency sub-bands *LH*, *HL*, and *HH* are the horizontal, vertical, and diagonal details of the image, and Ψ is the wavelet transform operator.

To integrate the high-frequency sub-bands, a 1 × 1 convolution is applied to preserve fine details while keeping the model lightweight. This integration is obtained through Equation (3):

(3) $$X_{Fused}=\emptyset \left(LH,HL,HH\right)$$

where ϕ is the convolution operation for combining information.

WCNN, by preserving multi-scale image information, simultaneously extracts textural details and overall brain structures. Because disease fluctuations occur at multiple scales, and wavelet analysis enhances the model’s accuracy in identifying these patterns in MRI images, this feature is crucial for accurate diagnosis.

In this module, the discrete wavelet transform is employed instead of conventional pooling to preserve both low- and high-frequency details of the image’s structural features.

### 3.3. The Coordinate Attention (CA) Module

**Figure 7:**
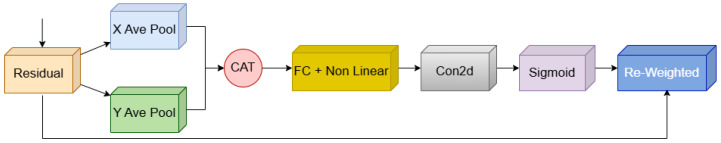
The p-CA module generates the attention weights by encoding spatial information in the horizontal and vertical directions and applies them to improve feature representation.

The CA module is designed to combine spatial and channelized information to better expose the features along the two spatial dimensions. In this method, global averaging is performed separately in the horizontal and vertical directions, resulting in two compressed vectors Figure 7. These vectors are converted into attention weights for each axis after passing through a dimensionality-reduction layer and a nonlinear mapping. A Sigmoid activation is applied to ensure the attention weights α_h_ and α_W_ are bounded between 0 and 1, stabilizing the reweighting of feature maps. The reweight feature is obtained through Equation (4):

(4) $$X_{output}=X\cdot \alpha_{h}\cdot \alpha_{W}$$

where α_h_ and α_W_ are the extracted attention coefficients for height and width. The advanced version of p-CA applies a Fully Connected layer rather than a convolution to model large-scale spatial dependencies. This change enables richer spatial encoding and enhances the model’s focus on sensitive areas in medical images. These attention modules, owing to their light weight and high performance, are particularly applicable to MRI image analysis, as disease-related changes are often subtle and region-specific.

### 3.4. Mid-Skip Connections

To improve information flow and preserve low-level and high-level features, mid-skip connections are maintained simultaneously. Unlike the classic U-Net, which establishes connections only between layers at the same level, or the Dense/Full-Skip structure that connects all layers, the Mid-Skip design establishes key connections between the low and high levels of the decoder. This setting enables simultaneous integration of local details and more abstract representations while reducing computational complexity and memory consumption by providing a more accurate reconstruction of multi-scale features in MRI images of PD.

### 3.5. Network Configuration

The Lite HGWA-Net comprises four encoders and four decoder layers connected through a bottleneck. Each encoder layer comprises a Hybrid Ghost Module and a WCNN to extract both spatial and frequency features while performing hierarchical downsampling. The number of channels in the encoder layers is 16, 24, 40, and 80, respectively, increasing to 160 in the bottleneck. The input MRI images, after resizing, are 192 × 192 × 1, and at each encoder layer, the spatial dimensions of the feature maps are halved. For downsampling, the WCNN block (DWT) from the Daubechies family (Db2) with a decomposition level of 1 is applied. The low-frequency LL sub-band output is preserved as the input to the next layer. The high-frequency sub-bands (LH, HL, and HH) are integrated through 1 × 1 convolutions to retain fine-grained details while keeping the model lightweight. In each encoder layer, downsampling is performed hierarchically to assure that spatial and frequency information is preserved across subsequent stages of the decoder and skip connections.

## 4. Experiments and Results

### 4.1. Databases

The Parkinson’s Progression Markers Initiative (PPMI) database, a publicly available, comprehensive resource for identifying biomarkers of PD progression, is employed here. Studies [39,40] focus on T2-weighted sMRI images, which are more appropriate for early PD diagnosis and more accurately depict iron accumulation, substantia nigra degeneration, and basal ganglia changes. This cohort contains 450 MRI images from patients with early PD and 103 images of the control group (50–79 years). To address class imbalance, 347 T2-weighted images from the IXI dataset control group are added to the dataset. All participants in both groups (patients and controls) are in good general health, with no history of neurological disorders or serious diseases. A Siemens Trio 3 Tesla MRI machine is applied for imaging. The data are available in NIfTI and DICOM formats, enabling accurate preprocessing and 3D processing. Ground-truth substantia nigra (SN) masks are available for the scans and are employed only to supervise the auxiliary SN localization branch. The primary labels (PD vs. healthy control) are used for the patient-level classification objective.

### 4.2. Preprocessing

MRI image preprocessing is a primary step in data analysis and directly affects the quality and accuracy of the results. As to the T2-weighted sMRI images, (1) the skull (Skull Stripping) and non-brain tissues like skin and skull bone are removed by applying standard tools, FSL BET [41], and Free Surfer [42]. The objective of this step is to focus on brain structures and reduce structural noise before the following steps, (2) noise reduction, and image artefact removal, which reduce the distortions caused by the imaging process. This step is essential for detecting slight pathological changes in the sensitive areas of the brain, like the substantia nigra, the vital contributor in PD, (3) Bias Field Correction is made through the N4ITK algorithm to correct the delusive changes in pixel intensity and prevent confusion with the fundamental changes in brain tissue [43], and (4) the images are registered to the MNI152 reference frame (MNI152NLin2009cAsym) for alignment with the standard brain space.

This process enables group analysis and comparison among all samples. Pixel intensity standardization is implemented to reduce inter-device variability and improve the performance of the learning model. To increase data diversity and reduce the risk of overfitting, data augmentation transformations [44], including rotation, translation, and minor geometric changes, are applied to the images. This set of preprocessing steps enhances data quality and provides a solid foundation for accurate feature extraction and effective modelling.

### 4.3. Measurement and Evaluation Criteria

The primary task in this study is patient-level classification (PD vs. healthy control). A set of standard criteria, including Accuracy, Recall, Precision, F1-Score, and mean Intersection over Union (mIoU), is applied to evaluate the performance of the proposed model, through Equations (5)–(8). These criteria are calculated from the four TP (true positive), TN (true negative), FP (false positive), and FN (false negative) values [45]. Due to the imbalanced nature of the dataset, *F*1-Score is considered the primary performance metric.

(5) $$Accuracy=\frac{TP+TN}{TP+TN+FP+FN}$$

(6) $$Recall=\frac{TP}{TP+FP}$$

(7) $$Precision=\frac{TP}{TP+FN}$$

(8) $$F1-Score=\frac{2*Precision*Recall}{Precision+Recall}$$

To further quantify the auxiliary segmentation branch, the overlap between the predicted SN mask and the ground-truth SN mask is evaluated. The Intersection over Union (IoU) is computed for each segmentation class through Equation (9):

(9) $${IoU}_{c}=\frac{{TP}_{c}}{{TP}_{c}+{FP}_{c}+{FN}_{c}}$$

The mean IoU (mIoU) is obtained by averaging the IoU over the SN and background classes through Equation (10).

(10) $$mIoU=\left(\frac{1}{N}\right)\times \sum_{c=1}^{N}{IoU}_{c}$$

### 4.4. Implementation Details

All experiments are conducted by applying PyTorch 2.2 on a laptop equipped with a 13th-generation Intel Core i9 processor and an NVIDIA RTX 4080 graphics card with 12 GB of memory. The models are trained using the Dice Loss function to improve texture-separation accuracy [46], and the network weights are updated through the Adam optimizer [47]. The batch size is 16, and the initial learning rate is 0.001. This learning rate decreases gradually by applying a Cosine Annealing Scheduler over 150 training epochs to assure stable and optimal model training [48]. Early stopping with a patience of 20 epochs based on the validation loss is necessary to prevent overfitting. A weight decay (L2 regularization) of 0.0001 is employed during training. Before entering the images into the model, all samples are resized to 192 × 192 pixels to match the network input structure. The model’s performance and generalizability are evaluated through a five-fold cross-validation. The data are divided into five equal parts, and in each iteration, one part is applied as the test set and the other four as the training set. The distribution of the data obtained from PPMI and IXI for each set is tabulated in Table 2. All experiments are conducted by applying a fixed random seed (42) to assure reproducibility.

The data partition in each set is 64% for training, 16% for validation, and 20% for testing, with class balance maintained across partitions (Table 2). The mean and SD of the evaluation indices from five iterations are calculated to provide an accurate view of the model performance.

### 4.5. Results

To evaluate the performance of the Lite HGWA-Net model, experiments are run on the PPMI dataset, and the results are compared with those of the basic and advanced architectures. The evaluation criteria consist of F1-score (considered the primary metric), Accuracy, Recall, Precision, mIoU, model parameters (Params), and computational complexity (FLOPs). The quantitative results from the comparison of model performance are tabulated in Table 3.

As observed in Table 3, the proposed model outperforms its counterparts across all evaluation metrics. This makes it an appropriate option for real-world environments and systems with limited hardware resources, particularly in medical imaging applications. This superior performance is attributed to the combination of Hybrid Ghost modules, wavelet-based downsampling, and attention mechanisms, which enable the model to efficiently extract discriminative features while minimizing computational cost. Compared with other state-of-the-art architectures, Lite HGWA-Net achieves a favourable balance between performance and computational efficiency, making it a practical choice for clinical applications.

**Figure 8:**
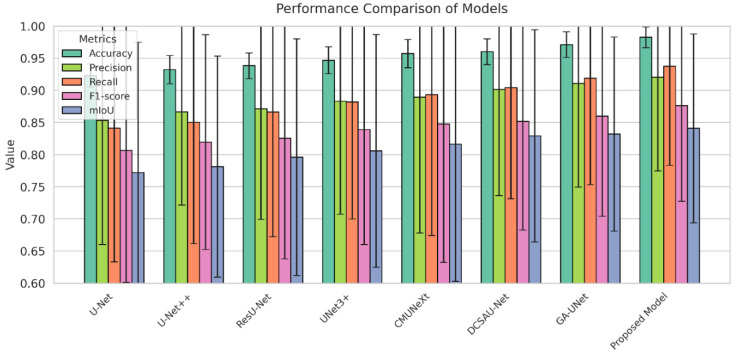
Comparison of different models’ performance by applying the Accuracy, Precision, Recall, F1-score, and mIoU metrics together with their standard errors.

**Figure 9:**
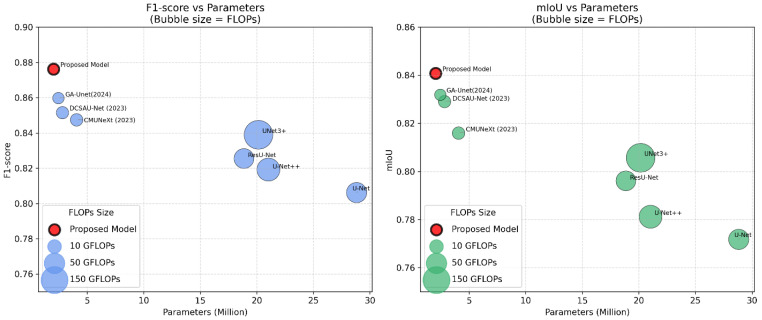
Comparison of this model with the leading architectures GA-UNet (2024), DCSAU-Net (2023), CMUNeXt (2023), and the base models (U-Net) in terms of F1-score and mIoU in terms of the number of parameters, where the size of the spots indicates the amount of FLOPs.

The performance of the models together with their standard errors is bar-charted in Figure 8. The comparison of the F1-score and mIoU of the models vs. the number of parameters and FLOPs is shown in Figure 9. The proposed mid-skip connections integrate local and abstract features efficiently. As shown in Table 3, the model achieves higher F1-score, Recall, and mIoU than UNet 3+, with fewer parameters and FLOPs, indicating reduced complexity.

The Lite HGWA-Net achieves higher values and lower fluctuations, indicating high stability and reliability Figure 8. This newly proposed model, with the fewest parameters and FLOPs, yields the highest F1-score and mIoU, indicating its lightweightness and computational efficiency.

Overall, the quantitative and qualitative results indicate that Lite HGWA-Net is a lightweight, efficient, and reliable solution for PD detection.

### 4.6. Ablation Study

**Figure 10:**
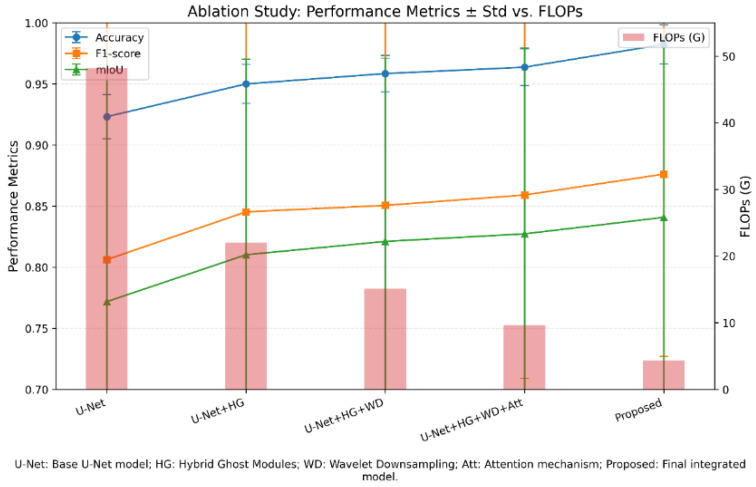
Results of the ablation study comparing the base U-Net model with the improved versions in successive stages. Accuracy, F1 score, and (mIoU) are plotted together with the SD. Number of Floating-Point Operations (FLOPs) b.

The performance of the basic U-Net model is compared with that of its improved versions at multiple successive stages. As observed in Table 4 and Figure 10, the base model (Base U-Net) has some limitations in terms of structures containing fine-grained resolution and computational cost reduction criteria, despite its acceptable accuracy.

As observed in Table 4 and Figure 10, in the first step, replacing the standard convolutional layers with Hybrid Ghost modules (a combination of GhostV2 and learned ensemble convolution) results in a considerable improvement in the Accuracy and Precision metrics. This improvement is due to GhostV2’s ability to extract rich features with lower computational cost and reduce redundancy in feature maps. The absence of a multi-resolution dimensionality-reduction mechanism at this stage limits the reduction in FLOPs relative to later versions.

In the second step, by replacing the traditional MaxPooling layers with Wavelet Downsampling, the computational complexity is reduced, and the accuracy and mIoU improve. These occurrences are due to the improved preservation of frequency and edge information during dimensionality reduction, which are essential for identifying fine-grained structures in medical imaging data, particularly in regions related to PD. The increase in the Recall metrics at this stage indicates that the model identifies true positives effectively.

In the third step, the addition of Attention Mechanisms and other optimizations leads to further improvement of the evaluation indices. Finally, this proposed model achieves the highest values for Accuracy, Recall, and F1-Score, while its parameter count and computational cost are considerably lower than those of the initial versions.

The results indicate that the synergistic effect among architectural optimization (Hybrid Ghost Modules), intelligent dimensionality reduction (Wavelet Downsampling), and attention mechanisms is essential for improving the performance of medical diagnosis models while maintaining computational efficiency. Together with the applied cross-validation strategy, the ablation experiments indicate that Lite HGWA-Net exhibits robust and consistent performance across different subsets of the dataset.

## 5. Discussion

In terms of classification performance, this proposed Lite HGWA-Net architecture outperforms its counterparts in early PD diagnosis from T2-weighted MRI images. The obtained F1-Score, accuracy, precision, recall, and mIoU of 0.8762, 0.9824, 0.9204, 0.9374, and 0.8408, respectively, exceed those of GA-UNet (0.8598) and DCSAU-Net (0.8291), and offer the lowest computational complexity (4.36 GFLOPs and 2.03 million parameters). These results indicate that the integration of Hybrid Ghost modules, Wavelet-based downsampling, and attention mechanisms improves both accuracy and efficiency in feature extraction and region localization simultaneously. The ablation study confirms the contribution of each component. Adding Hybrid Ghost modules to the base U-Net increases F1-Score from 0.8062 to 0.8453; further improving it to 0.8506 by adding wavelet downsampling underscores the importance of preserving frequency and texture information, particularly for detecting subtle pathological changes in the substantia nigra. When the focus is on more disease-related regions, the attention mechanism improves performance to 0.8591. The final model, in which all components are integrated, is the best-performing, with a considerable reduction in computational cost compared with available deep architectures.

Compared with available models like UNet++ and ResU-Net, this newly proposed model architecture achieves higher accuracy and a substantial reduction in FLOPs and metrics. This feature makes Lite HGWA-Net an appropriate candidate for resource-constrained clinical environments or for real-time applications, like in situ diagnosis or integration into portable imaging devices. Applying the wavelet transform for downsampling here aligns with previous studies, where the benefits of multi-resolution analysis in medical imaging are evident. The results here extend this concept, revealing that combining wavelets with lightweight convolutional blocks and attention mechanisms can achieve more advanced performance in MRI analysis of PD.

No process is without its limitations. The data applied here are from the PPMI database, which may limit the model’s generalizability to other MRI protocols or populations. To fully evaluate the robustness and generalizability of Lite HGWA-Net, external validation on multi-centre datasets is necessary and is considered a key direction for future studies. Moreover, although T2-weighted images are effective for visualizing the substantia nigra, multimodal MRI sequences, like SWI and DTI, can provide complementary information to enhance diagnostic robustness. The focus of the upcoming studies should be on validating Lite HGWA-Net on multicenter datasets, assessing its applicability to other neurodegenerative disorders, and exploring domain-adaptation strategies for generalization across scanners. Overall, Lite HGWA-Net demonstrates that a well-designed combination of lightweight convolutional blocks, frequency-preserving downsampling, and attention-based feature refinement can achieve high diagnostic accuracy while maintaining computational efficiency. This makes the model a promising tool in assisting clinicians in early diagnosis of PD.

## 6. Conclusions

The Lite HGWA-Net achieved high diagnostic performance for early Parkinson’s disease detection from T2-weighted MRI images, yielding an F1-Score of 0.8762 while maintaining a lightweight, optimized architecture with a parameter number of 2.03 M and a computational cost of 4.36 GB. By addressing the gap in accessible, resource-efficient models for early PD diagnosis, this study advances the field by demonstrating that the combination of Hybrid Ghost modules, wavelet-based downsampling, and attention mechanisms can retain high-fidelity discrimination without the computational burden imposed by conventional architectures. This convergence of high F1-Score, competitive performance across metrics, and sharply reduced computational demand provides an immediately deployable option for clinical environments with limited computational resources, supporting workflows that require fast and reliable assessment of disease-related brain structures. The next step is to test the model across diverse imaging settings to assure consistent behaviour and broaden its applicability. In the future, the model will be validated on multicenter datasets, and additional biomarkers as well as multimodal imaging data will be integrated to further improve its performance and generalizability.

## Figures and Tables

**Table 2:** Data distribution in each set (training, validation, and testing).

Group	Total	Training (64%)	Validation (16%)	Test (20%)
PD patients	450	288	72	90
Control group	103	66	17	20
Healthy controls	347	222	56	69
Total	900	576	145	179

**Table 3:** Quantitative results of the different models evaluated on the PPMI dataset SN mIoU (aux. seg.) reports the mean IoU of the substantia nigra segmentation branch, while the remaining metrics correspond to the primary patient-level classification task.

Model	Accuracy	Precision	Recall	F1-Score	SN mIoU (aux. seg.)	FLOPs(G)	Params (M)
U-Net [49]	0.9232 ± 0.018	0.8532 ± 0.193	0.8412 ± 0.208	0.8062 ± 0.205	0.7718 ± 0.203	48.21	28.81
U-Net + + [50]	0.9321 ± 0.022	0.8664 ± 0.145	0.8504 ± 0.189	0.8193 ± 0.167	0.7812 ± 0.172	79.86	21.02
ResU-Net [51]	0.9382 ± 0.020	0.8712 ± 0.172	0.8661 ± 0.194	0.8256 ± 0.188	0.7961 ± 0.184	44.37	18.84
UNet3 + [52]	0.9465 ± 0.021	0.8832 ± 0.176	0.8817 ± 0.182	0.8390 ± 0.179	0.8057 ± 0.181	195.74	20.14
CMUNeXt [53]	0.9571 ± 0.022	0.8891 ± 0.211	0.8932 ± 0.219	0.8475 ± 0.215	0.8160 ± 0.213	7.14	4.06
DCSAU-Net [54]	0.9601 ± 0.020	0.9013 ± 0.165	0.9041 ± 0.173	0.8516 ± 0.169	0.8291 ± 0.165	6.68	2.81
GA-U-Net [55]	0.9710 ± 0.020	0.9106 ± 0.161	0.9189 ± 0.166	0.8598 ± 0.156	0.8319 ± 0.151	4.96	2.45
This Model	0.9824 ± 0.016	0.9204 ± 0.146	0.9374 ± 0.154	0.8762 ± 0.149	0.8408 ± 0.147	4.36	2.03

**Table 4:** Comparison of the base U-Net model and its improved versions based on the (Acc), (Prec), (Rec), (mIoU), (FLOPs), and (Params) primary criteria.

Model	Accuracy	Precision	Recall	F1-Score	SN mIoU (aux. seg.)	FLOPs(G)	Params (M)
U-Net [49]	0.9232 ± 0.018	0.8532 ± 0.193	0.8412 ± 0.208	0.8062 ± 0.205	0.7718 ± 0.203	48.21	28.81
UNet + HG	0.9500 ± 0.016	0.8875 ± 0.165	0.8849 ± 0.168	0.8453 ± 0.162	0.8102 ± 0.160	22.01	13.02
UNet + HG + WD	0.9585 ± 0.015	0.8950 ± 0.155	0.9001 ± 0.154	0.8506 ± 0.158	0.8211 ± 0.150	15.12	13.00
UNet + HG + WD + Att	0.9637 ± 0.015	0.9026 ± 0.153	0.9147 ± 0.159	0.8591 ± 0.150	0.8273 ± 0.152	9.67	6.24
This Model	0.9824 ± 0.016	0.9204 ± 0.146	0.9374 ± 0.154	0.8762 ± 0.149	0.8408 ± 0.147	4.36	2.03

HG: Hybrid Ghost Modules; WD: Wavelet Downsampling; Att: Attention mechanism; This Model: Final integrated model.

## Data Availability

The datasets are publicly available from the PPMI database (https://www.ppmi-info.org, accessed on 16 October 2024) and the IXI dataset (IXI Dataset—Brain Development, accessed on 7 November 2024).

## Abbreviations

The following abbreviations are employed in this manuscript:

HGWA	Hybrid Ghost Wavelet Attention
CNN	Convolutional Neural Network
PD	Parkinson’s Disease
MRI	Magnetic Resonance Imaging
AI	Artificial Intelligence
MSA	Multiple System Atrophy
WCNN	Wavelet-based CNN
LGC	Learned Group Convolution
SN	Substantia Nigra
DLGC	Dynamic Learning Group Convolution
DFCA	Decomposed Fully Connected Attention
DWT	Discrete Wavelet Transform
CA	Coordinate Attention
sMRI	Structural MRI
SN mIoU (aux. seg.)	Mean Intersection over Union
TP	True Positive
TN	True Negative
FP	False Positive
FN	False Negative
HC	Healthy Control
